# EYA2 Correlates With Clinico-Pathological Features of Breast Cancer, Promotes Tumor Proliferation, and Predicts Poor Survival

**DOI:** 10.3389/fonc.2019.00026

**Published:** 2019-01-29

**Authors:** Hanxiao Xu, Ying Jiao, Ming Yi, Weiheng Zhao, Kongming Wu

**Affiliations:** Department of Oncology, Tongji Hospital of Tongji Medical College, Huazhong University of Science and Technology, Wuhan, China

**Keywords:** breast cancer, EYA2, tumor grade, molecular subtypes, proliferation, epithelial-mesenchymal transition, cancer stem cells, prognosis

## Abstract

Eyes absent homolog 2 (EYA2), a transcriptional activator, is pivotal for organ development, but aberrant regulation of EYA2 has been reported in multiple human tumors. However, the role of EYA2 in breast cancer is still lack of full understanding. To explore the biological significance of EYA2 in breast cancer, we conducted data analysis on public breast cancer datasets, and performed immunohistochemistry (IHC) analysis, colony-forming unit assays, EdU assay, western blotting, and immunofluorescence (IF). Meta-analysis showed that *EYA2* mRNA expression was correlated with tumor grade, the status of estrogen receptor (ER), progesterone receptor (PR), and human epidermal growth factor receptor 2 (HER2). IHC analysis displayed that EYA2 protein abundance was inversely associated with the status of ER and PR, and enriched in triple-negative breast cancer in comparison with luminal-type tumors. Additionally, correlation analysis reflected that *EYA2* mRNA was negatively correlated with luminal markers, and positively associated with markers of basal cells, epithelial-mesenchymal transition and cancer stem cells. Clone-forming assay and EdU experiment showed that EYA2 overexpression enhanced proliferation of breast cancer cells. Results from western blotting and IF displayed that overexpression of EYA2 up-regulated the protein abundance of proliferation markers. Importantly, survival analysis indicated that higher *EYA2* mRNA level predicted worse overall survival, relapse-free survival and metastasis-free survival among whole enrolled breast cancer patients. Collectively, EYA2 was closely correlated with clinico-pathological characteristics, and served as a proliferation stimulator for breast cancer cells and an unfavorable prognostic element for breast cancer patients, suggesting that EYA2 is involved in the progression of breast carcinoma.

## Introduction

Breast cancer is the leading cancer type in women and poses a major threat to public health worldwide (1). During the past decades, many efforts have been exerted to better management of this tumor type (2–7). However, current understanding of molecular mechanisms for this highly heterogenous disease is not fully clear (8). Identification of key molecules that promote and maintain malignant conversion facilitates comprehensive understanding of breast tumor biology, ultimately contributing to novel potential targets for drugs and better management of breast carcinoma (3, 9, 10).

Retinal determination gene network (RDGN), mainly including dachshund (DACH), sine oculis (SIX) and eyes absent (EYA), governs organ development (11). EYA family members, including EYA1, EYA2, EYA3, and EYA4, contain a C-terminal 271 amino-acid region for the interaction with other proteins and a N-terminal domain responsible for the innate immune response with inherent threonine phosphorylation activity (12–14). Recently, the dysregulation of EYA2 has been reported to be involved in several human cancers (15–18). For instance, EYA2 expression was enhanced in multiple tumors in comparison with corresponding normal tissues (16, 17). EYA2 can promote tumor growth in diverse tumor types (16–19). In human astrocytoma, EYA2 promoted cell cycle progression of tumor cells via the up-regulation of cyclin D1 and cyclin E (17). Knockdown of EYA2 by siRNA reduced the proliferation through cell cycle G1 block and enhanced the apoptosis of lung adenocarcinoma cells (20). On the contrary, one previous research demonstrated that EYA2 overexpression was an unfavorable molecule for tumor growth of pancreatic adenocarcinoma in orthotopic models (21). In addition, EYA2 can also contribute to tumor invasion and metastasis for some cancer types, including breast cancer (18), lung adenocarcinoma (22), and astrocytoma (17). Several mechanisms might underlie the role of EYA2 in tumor invasion, including the activation of ERK signaling (17) and the promotion of epithelial-mesenchymal transition (EMT) (18). The association between EYA2 and clinical outcomes is controversial. High EYA2 level has been demonstrated to be a negative element for prognosis in lung cancer (16), while EYA2 predicted better clinical outcomes in colorectal cancer (15) and pancreatic cancer (21).

A recent study has demonstrated that microRNA-338-3p/EYA2 axis led to epidermal growth factor receptor (EGFR)-induced tumor growth and lung metastasis (18). However, the role of EYA2 in breast cancer remains to be further explored. Herein, we performed a meta-analysis of public available breast cancer gene expression omnibus (GEO) datasets to further detect the differential expression of *EYA2* in normal vs. breast tumors, and to explore the association between *EYA2* and tumor differentiation, the status of estrogen receptor (ER), progesterone receptor (PR) and human epidermal growth factor receptor 2 (HER2) as well as molecular subtypes at mRNA level. Immunohistochemistry (IHC) analysis on tissue microarray (TMA) was conducted to explore the correlation between EYA2 and the status of ER and PR as well as molecular subtypes at protein level. Furthermore, correlation analysis of GSE25066 was performed to explore the association between *EYA2* and markers of luminal, triple-negative breast cancer (TNBC), EMT, cancer stem cells (CSCs) as well as the cell cycle-related gene. Besides, we conducted colony-forming unit assays, EdU experiments, western blotting, and immunofluorescence (IF) to evaluate the role of EYA2 in tumor proliferation and explore EYA2 regulated genes Finally, we employed the Kaplan-Meier Plotter platform to explore the role of *EYA2* in the prognosis of breast cancer patients.

## Materials and Methods

### IHC Staining and Quantification Evaluation

One commercially available TMA slide (HBre-Duc140Sur-01, Shanghai Outdo Biotech Co., Ltd.) was purchased for IHC analysis, which contained histologically confirmed breast cancer tissues with clinico-pathological information, such as tumor grade, clinical stage and the status of ER, PR, and HER2 in IHC (Table 1). Breast tumors with positive status of ER or PR belong to luminal-type, and tumors that do not express ER, PR, and HER2 are TNBC. Due to tissue shedding of 15 cases, the number of actually available tissue points was 125. To evaluate the protein abundance of EYA2 in ER– vs. ER+, PR– vs. PR+, and luminal-type vs. TNBC tissues as well as the prognostic value among breast cancer population, IHC analysis was conducted with a standard protocol described previously (23). The specific primary antibody against EYA2 (ab95875, Abcam) was utilized for IHC at a dilution of 1:100.

**Table 1:** Clinico-pathological information of patients in tissue microarray (HBre-Duc140Sur-01).

**Variables**	**Patient number**
**GRADE**	
1	9
1–2	21
2	87
2–3	6
3	1
**TNM**	
I	8
II	71
III	44
**ER**	
ER–	42
ER+	76
**PR**	
PR–	49
PR+	68
**HER2**	
HER2–	81
HER2+	13
**MOLECULAR SUBTYPES**	
Luminal	81
HER2-enriched	3
TNBC	23

*ER, estrogen receptor; PR, progesterone receptor; HER2, human epidermal growth factor receptor 2; TNBC, triple-negative breast cancer*.

Two experienced pathologists performed IHC scoring independently with no prior knowledge of the clinico-pathological information. The multiplication of intensity and proportion of positive-staining tumor cells was exploited to quantify the protein levels of EYA2 according to a standard protocol as described previously (24).

### Meta-Analysis

We carried out a meta-analysis of 14 relevant GEO breast cancer databases for the mRNA expression of *EYA2* available in ArrayExpress (Table 2) (25–38). Cutoff value for *EYA2* was median expression. The STATA software package (version 12.0) (Stata Corp LP, College Station, TX, USA) was employed to perform the meta-analysis. Odds ratio (OR) and 95% confidence intervals (95% CIs) were used to evaluate the association between *EYA2* mRNA and clinico-pathological factors. Overall survival (OS), relapse-free survival (RFS) and metastasis-free survival (MFS) were assessed by hazard ratio (HR) and 95% CIs. Heterogeneity of publication was evaluated by means of the inconsistency index I^2^.

**Table 2:** A list of enrolled GEO datasets in meta-analysis.

**GEO datasets**	**First author**	**Year**	**Number of patients**	**References**
GSE10885	Hennessy BT	2009	89	(25)
GSE17907	Sircoulomb F	2010	51	(26)
GSE20711	Dedeurwaerder S	2011	88	(27)
GSE39004	Terunuma A	2014	61	(28)
GSE25066	Hatzis C	2011	508	(29)
GSE45255	Nagalla S	2013	139	(30)
GSE6532	Loi S	2010	327	(31)
GSE7390	Desmedt C	2007	198	(32)
GSE58644	Tofigh A	2014	321	(33)
GSE16446	Desmedt C	2011	120	(34)
GSE2603	Minn AJ	2005	99	(35)
GSE2034	Wang Y	2005	286	(36)
GSE1456	Pawitan Y	2005	159	(37)
GSE20685	Kao KJ	2011	327	(38)

*GEO, gene expression omnibus*.

### Correlation Analysis of Gene Expression Data

GEO dataset GSE25066, containing 508 breast carcinoma patients, was analyzed to evaluate the correlation between *EYA2* mRNA level and the mRNA expression of *ESR1, PGR, forkhead box A1* (*FOXA1), keratin 5 (KRT5), KRT6B, EGFR, SNAI2, Y-box binding protein 1* (*YBX1*), *kruppel like factor 5 (KLF5), sex determining region Y-box 10 (SOX10), CCNE1*, and *DACH1*.

### Cell Culture and Establishment of EYA2 Stable Cell Lines

The breast cancer cell lines (MCF-7 and MDA-MB-231) were cultured in high-glucose Dulbecco's modified Eagle's medium supplemented with 10% fetal bovine serum (Life Technologies, Inc.) under the condition of 37°C and 5% CO_2_ in a humidified incubator. Retrovirus expression vector for *EYA2* (*MSCV-EYA2*, #49265) was purchased from Addgene. Human embryonic kidney 293T cells were transfected with the combination of expression vector or control vector with human package plasmids by Lipofectamine™ 2000 (Invitrogen, Carlsbad CA, USA). After transfection, the viral supernatant was harvested and filtered to infect MCF-7 and MDA-MB-231 cells for three times as described previously (39). EYA2 stable cells were selected by green fluorescent protein.

### Western Blotting

Protein extraction from MCF-7 and MDA-MB-231 cells and western blotting were conducted according to a standard protocol previously described (24). The primary antibodies used were as following: EYA2 (ab95875, Abcam), YBX1 (sc-101198, Santa Cruz), EGFR (sc-03, Santa Cruz), cyclin E (sc-25303, Santa Cruz), PCNA (sc-7907, Santa Cruz), and β-actin (sc-47778, Santa Cruz).

### Cellular IF

IF staining was performed based on published methods (39, 40). Primary antibodies were used at 1:150 dilution as following: EYA2 (ab95875, Abcam), YBX1 (sc-101198, Santa Cruz), EGFR (sc-03, Santa Cruz) and PCNA (sc-7907, Santa Cruz). The goat anti-mouse and the goat anti-rabbit secondary antibodies (Alexa Fluor-568) were both used at 1:300. Cell nuclei were stained with Hoechst 33342 at the dilution of 1:1,000.

### Colony-Forming Unit Assays

The colony-forming assay was performed as previously described (41). Breast cancer cell lines (MCF-7 and MDA-MB-231) with EYA2 overexpression or vector control were seeded in 3.5 cm dishes (3 × 10^3^ cells). Culture medium was changed every three days. At day 12, cells were fixed with 4% paraformaldehyde for 20 min and stained with 0.5% crystal violet for visualization.

### EdU Assay

Cell-Light^TM^ EdU Apollo567 In Vitro Kit (C10310-1, RIBOBIO, Guangzhou) was purchased for EdU experiment. Firstly, cells were seeded in 24-well plates at a density of 3 × 10^3^ per well and incubated for 2 days. Secondly, 250 μl of EdU medium was added to each well and incubated for 2.5 h. Next, cells were fixed with 4% paraformaldehyde in phosphate buffered saline (PBS) for 20 min and subsequently added glycine solution (2 mg/ml) to neutralize aldehyde group. After increasing cell membrane permeability with 0.5% TritonX-100 in PBS, Apollo dyeing reaction solution was added and incubated in dark place at room temperature for 30 min. Then, dye nucleus with Hoechst 33342 in dark place at room temperature for 30 min. Finally, fluorescence microscope was employed for observation and counting.

### Statistical Analysis

The Student's *t*-test was applied to evaluate the differences in groups. Correlation analysis was performed using Statistical Product and Service Solutions (SPSS) 20 statistical software (SPSS Inc., Chicago, IL, USA). A two-tailed *p*-value < 0.05 was considered statistically significant.

## Results

### EYA2 mRNA Expression in Normal Breast and Breast Tumors

**Figure 1:**
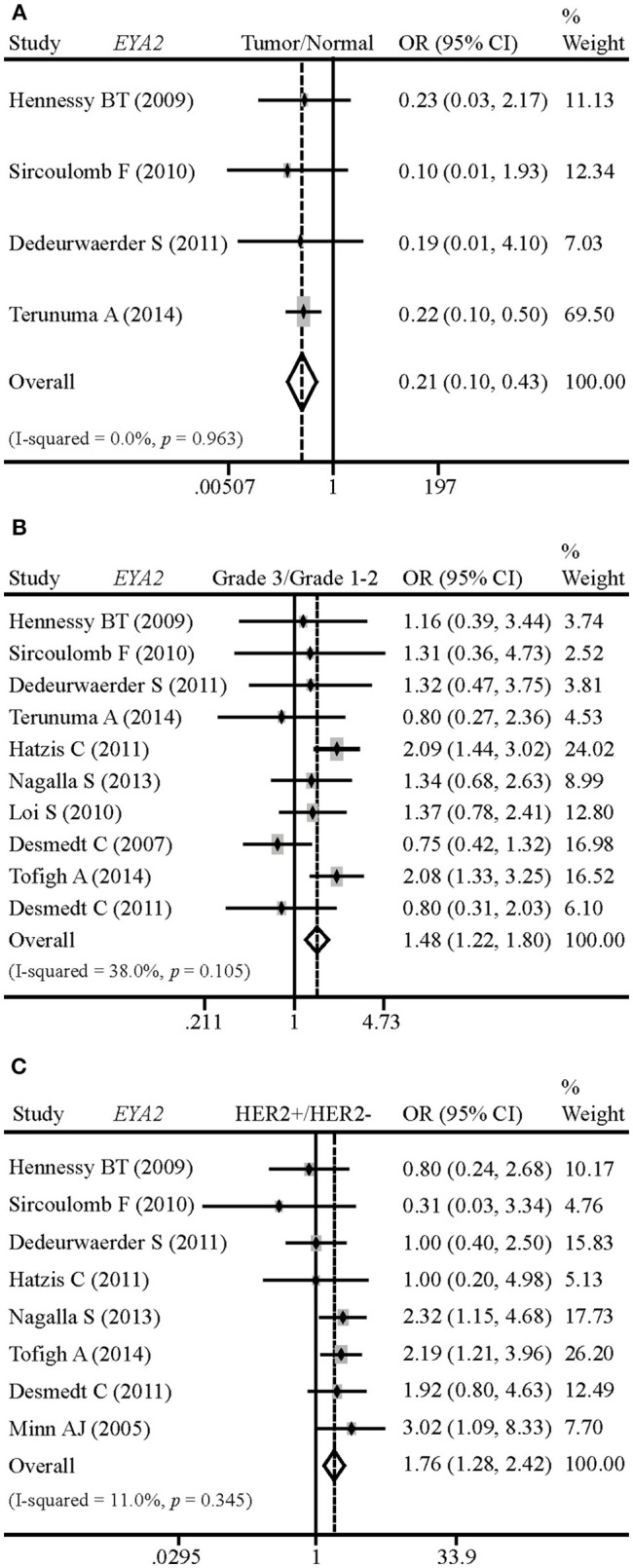
Differences of *EYA2* mRNA in breast tumors vs. normal breast, and the correlation between *EYA2* mRNA and tumor grade and HER2 status. *EYA2* mRNA level was remarkably lower in cancerous tissues than non-cancerous tissues **(A)**. EYA2 mRNA expression was significantly higher in high-grade cancer tissues **(B)** and HER2+ tumors **(C)** in comparison with low-grade and HER2− tumors, respectively.

In order to evaluate *EYA2* mRNA level in normal breast tissues vs. malignant tissues, grade 3 vs. grade 1–2 tumors, and HER2– vs. HER2+ tumors, we conducted a comprehensive meta-analysis of 14 GSE datasets. The results showed that *EYA2* mRNA expression was remarkably lower in cancerous tissues than in non-cancerous tissues [OR: 0.21 (0.10–0.43), *I*^2^ = 0.0%; Figure 1A]. *EYA2* mRNA level was significantly higher in high-grade cancer tissues [OR: 1.48 (1.22–1.80), *I*^2^ = 38.0%; Figure 1B) and HER2+ tumors [OR: 1.76 (1.28–2.42), *I*^2^ = 11.0%; Figure 1C] in comparison with low-grade tumor tissues and HER2– tumor tissues, respectively.

### Correlation Between EYA2 Expression and the Status of ER and PR

**Figure 2:**
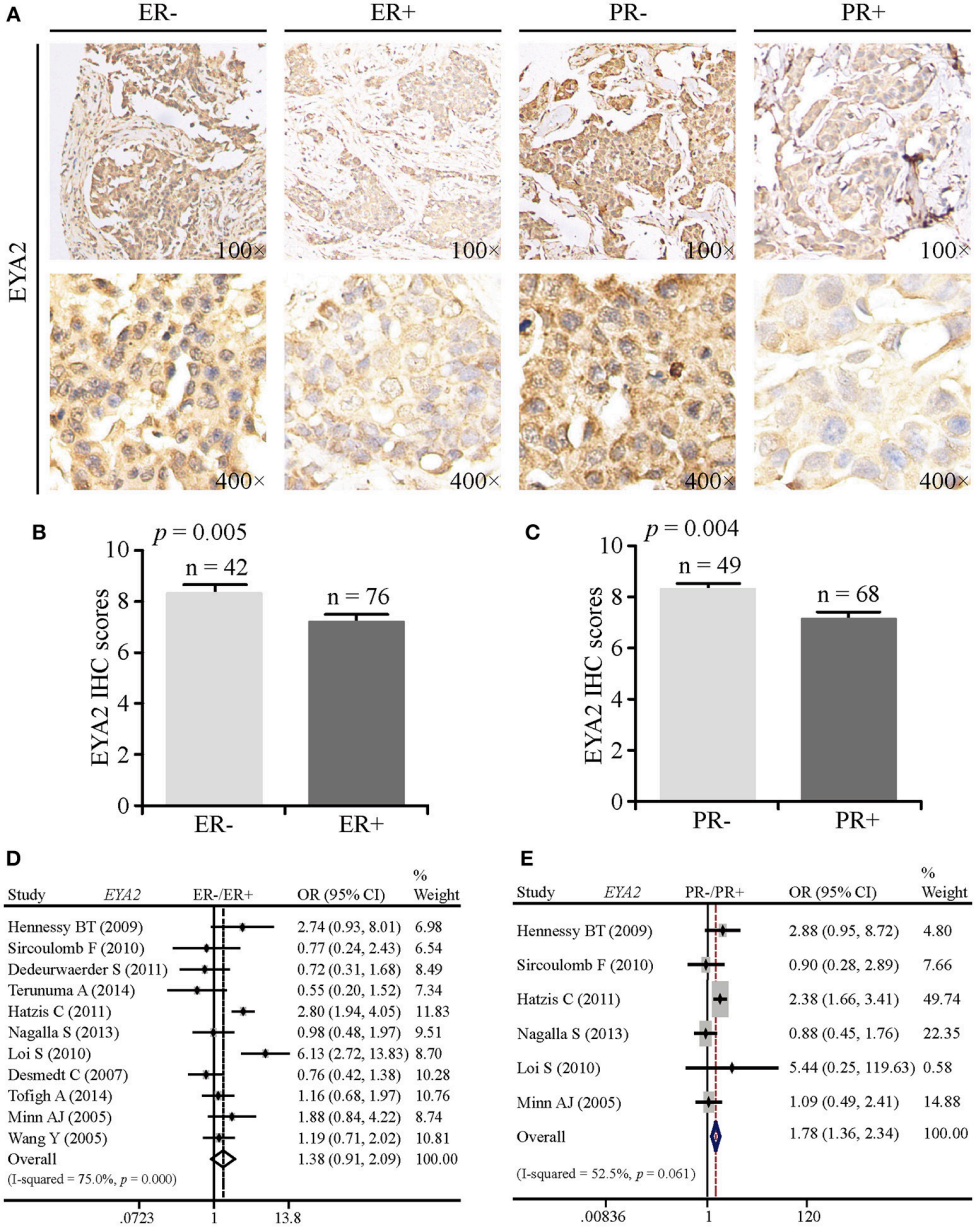
The correlation between EYA2 expression and the status of ER and PR. **(A)** Representative images of IHC staining for ER− vs. ER+ and PR− vs. PR+ were shown. Statistical analysis of IHC scores indicated that protein abundance of EYA2 was significantly higher in ER− **(B)** or PR− **(C)** in comparison with ER+ or PR+ cancer tissues, respectively. The results were shown as mean + SEM. **(D)** There was no significant difference in the EYA2 mRNA level between ER− and ER+ cancer tissues. **(E)** EYA2 mRNA expression was significantly enhanced in PR− tumors in comparison with PR+ breast cancer.

To compare EYA2 protein abundance in ER– vs. ER+ and PR– vs. PR+, we analyzed a TMA containing 125 informative cancer tissue points by IHC. EYA2 was majorly detected in the cytoplasm of breast cancer cells. Representative images of IHC staining were shown in Figure 2A. Next, IHC scores by using semi-quantitative criteria were also examined. The results indicated that protein abundance of EYA2 was significantly higher in ER– (*p* = 0.005) (Figure 2B) or PR– (*p* = 0.004) (Figure 2C) in comparison with ER+ or PR+ cancer tissues, respectively.

To assess whether the mRNA expression of *EYA2* is consistent with the protein expression, meta-analysis was performed. There was an increasing tendency of *EYA2* mRNA expression in ER– tumors in comparison with ER+ tumors. However, the difference was not statistically significant [OR: 1.38 (0.91–2.09), *I*^2^ = 75.0%; Figure 2D]. The mRNA expression of *EYA2* was higher in PR– tumors than PR+ tumors [OR: 1.78 (1.36–2.34), *I*^2^ = 52.5%; Figure 2E].

### EYA2 Is Associated With Molecular Subtypes of Breast Cancer

**Figure 3:**
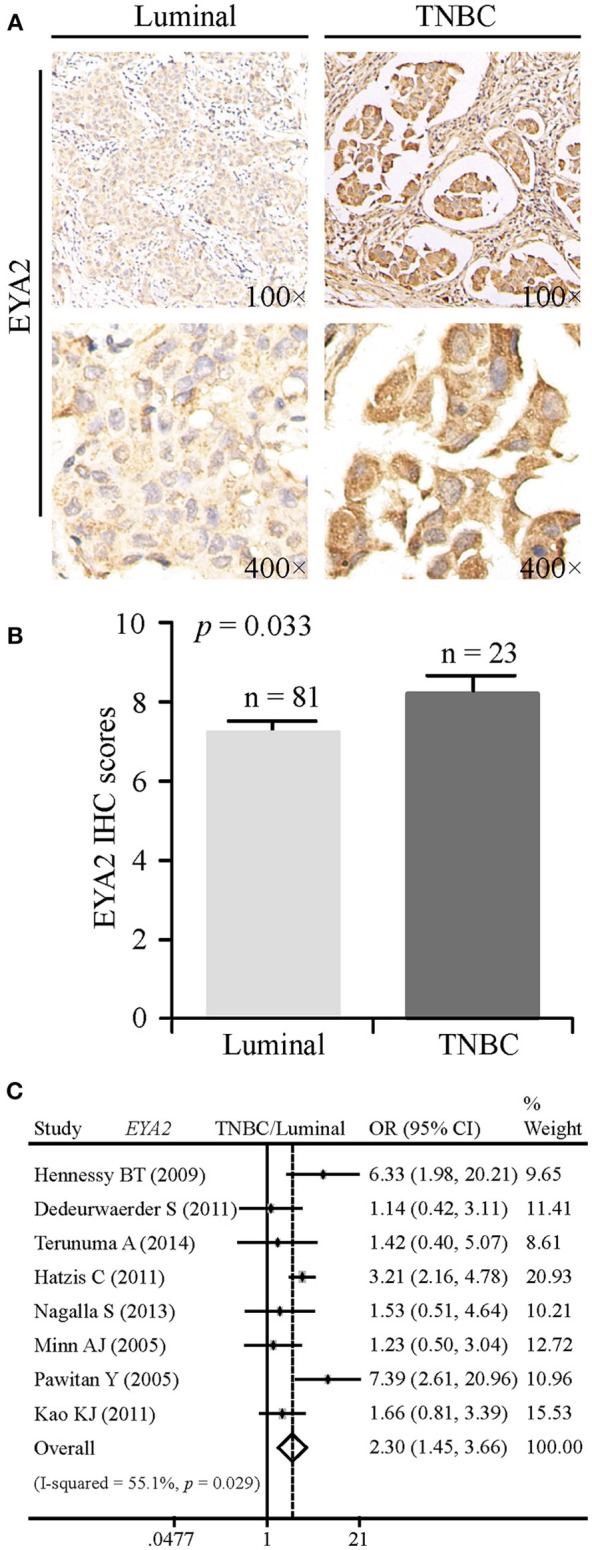
EYA2 is associated with molecular subtypes of breast cancer. **(A)** Representative images of IHC staining for luminal-type and TNBC breast cancer tissues were showed. **(B)** IHC scores revealed that higher level of EYA2 protein was significantly enhanced in TNBC tissues in comparison with luminal-type tissues, which was displayed as mean + SEM. **(C)** The mRNA level of *EYA2* in TNBC was much higher than that in luminal-type.

To elucidate whether there was any association between EYA2 protein abundance and molecular subtypes, we conducted IHC analysis on the TMA. Representative images of IHC staining for luminal-type and TNBC tissues were, respectively, showed in Figure 3A. Statistical analysis on IHC scores revealed that EYA2 protein level was significantly enhanced in TNBC tissues in comparison with luminal-type tissues (*p* = 0.033) (Figure 3B).

In order to explore whether the mRNA level of *EYA2* is consistent with the protein expression in distinct molecular subtypes, meta-analysis was performed. The mRNA level of *EYA2* in TNBC was much higher than in luminal-type [OR: 2.30 (1.45–3.66), *I*^2^ = 55.1%; Figure 3C]. We further evaluated the EYA2 mRNA expression in the Cancer Genome Atlas breast cancer dataset downloaded from UCSC Xena. We found that *EYA2* expression was significantly higher in basal-like tumors than luminal-type cancer tissues (*p* < 0.0001) (Supplementary Figure 1A), which was consistent with the results from our meta-analysis. Collectively, we drew a conclusion that EYA2 was enriched in TNBC tissues in comparison with luminal-type tissues at both protein and mRNA levels.

### The Correlations Between EYA2 mRNA and the Markers of Luminal, TNBC, Mesenchymal, CSCs as Well as Cancer-Related Genes

**Figure 4:**
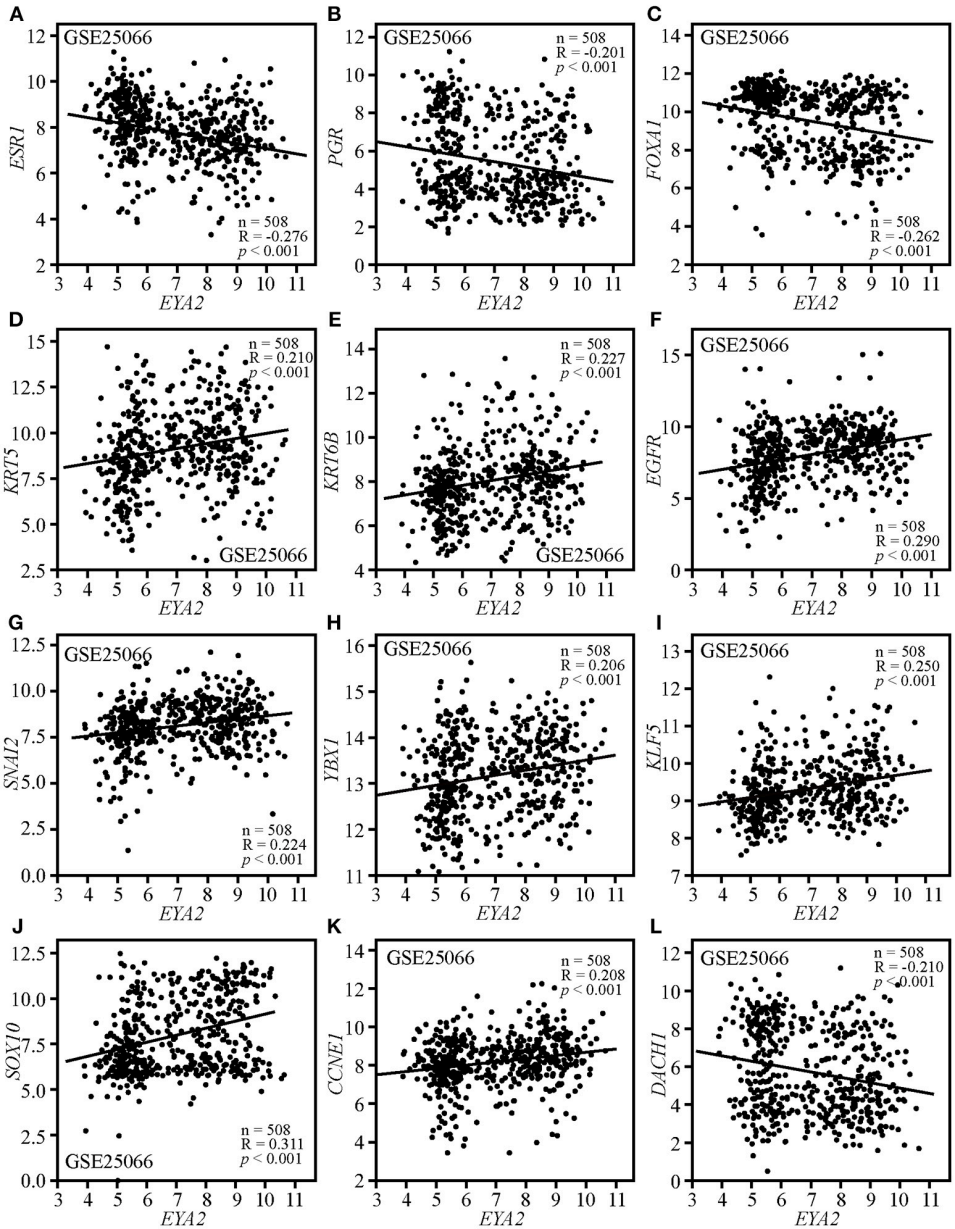
The correlations between *EYA2* with the markers of luminal, TNBC, mesenchymal, CSCs, proliferation as well as *DACH1*. *EYA2* mRNA level was negatively associated with the mRNA expression of *ESR1* **(A)**, *PGR* **(B)**, *FOXA1* **(C)**, and *DACH1* **(L)**, while it was positively correlated with *KRT5* **(D)**, *KRT6B* **(E)**, *EGFR* **(F)**, *SNAI2* **(G)**, *YBX1* **(H)**, *KLF5* **(I)**, *SOX10* **(J)**, and *CCNE1* **(K)**.

Correlation analysis was conducted on GSE25066, containing 508 breast cancer patients with distinct molecular subtypes. The results showed that *EYA2* mRNA level was negatively associated with the mRNA expression of *ESR1* (*R* = −0.276, *p* < 0.001; Figure 4A), *PGR* (*R* = −0.201, *p* < 0.001; Figure 4B) and *FOXA1* (*R* = −0.262, *p* < 0.001; Figure 4C), while it was positively correlated with *KRT5* (*R* = 0.210, *p* < 0.001; Figure 4D), *KRT6B* (*R* = 0.227, *p* < 0.001; Figure 4E), and *EGFR* (*R* = 0.290, *p* < 0.001; Figure 4F). In addition, the mRNA expression of *EYA2* was positively associated with *SNAI2* (*R* = 0.224, *p* < 0.001; Figure 4G), *YBX1* (*R* = 0.206, *p* < 0.001; Figure 4H), *KLF5* (*R* = 0.250, *p* < 0.001; Figure 4I), *SOX10* (*R* = 0.311, *p* < 0.001; Figure 4J), and *CCNE1* (*R* = 0.208, *p* < 0.001; Figure 4K), while *EYA2* mRNA was inversely correlated with RDGN gene *DACH1* (*R* = −0.210, *p* < 0.001; Figure 4L).

### EYA2 Promotes the Proliferation of Breast Cancer Cells With the Regulation of PCNA, EGFR, and YBX1

**Figure 5:**
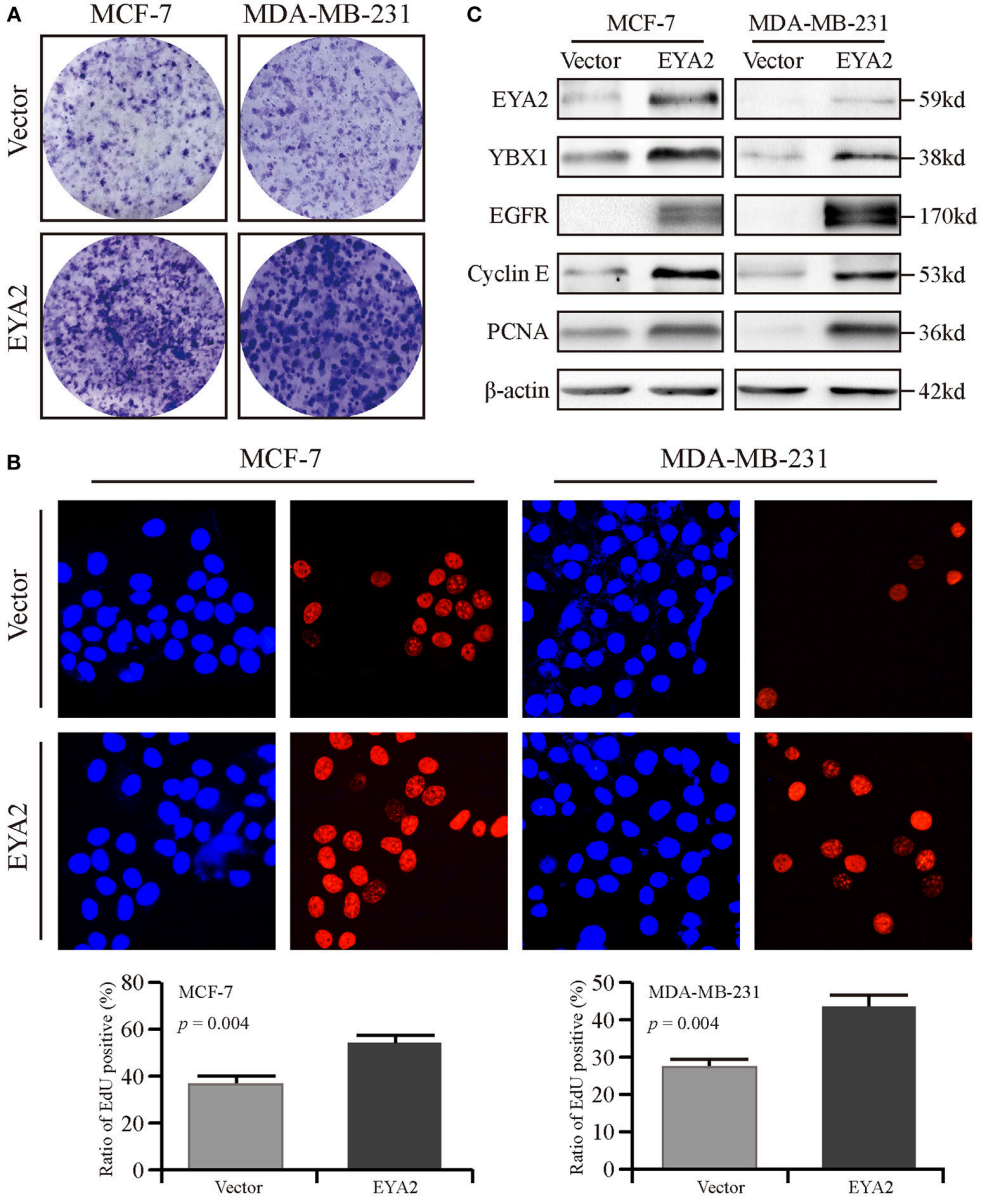
EYA2 promotes the proliferation of breast cancer cells. **(A)** Colony-forming unit assays showed that both MCF-7 and MDA-MB-231 with EYA2 overexpression formed more clones with the same number of initiating tumor cells than the controls. **(B)** EdU cell proliferation assay displayed that the ratio of proliferative cells is much higher among EYA2-overexpressing cancer cells than empty vector controls for both MCF-7 and MDA-MB-231. **(C)** Western blotting showed that EYA2 overexpression contributed to up-regulation of the cancer stem cell marker YBX1, proliferative markers of EGFR, cyclin E, and PCNA in both MCF-7 and MDA-MB-231 at protein level.

**Figure 6:**
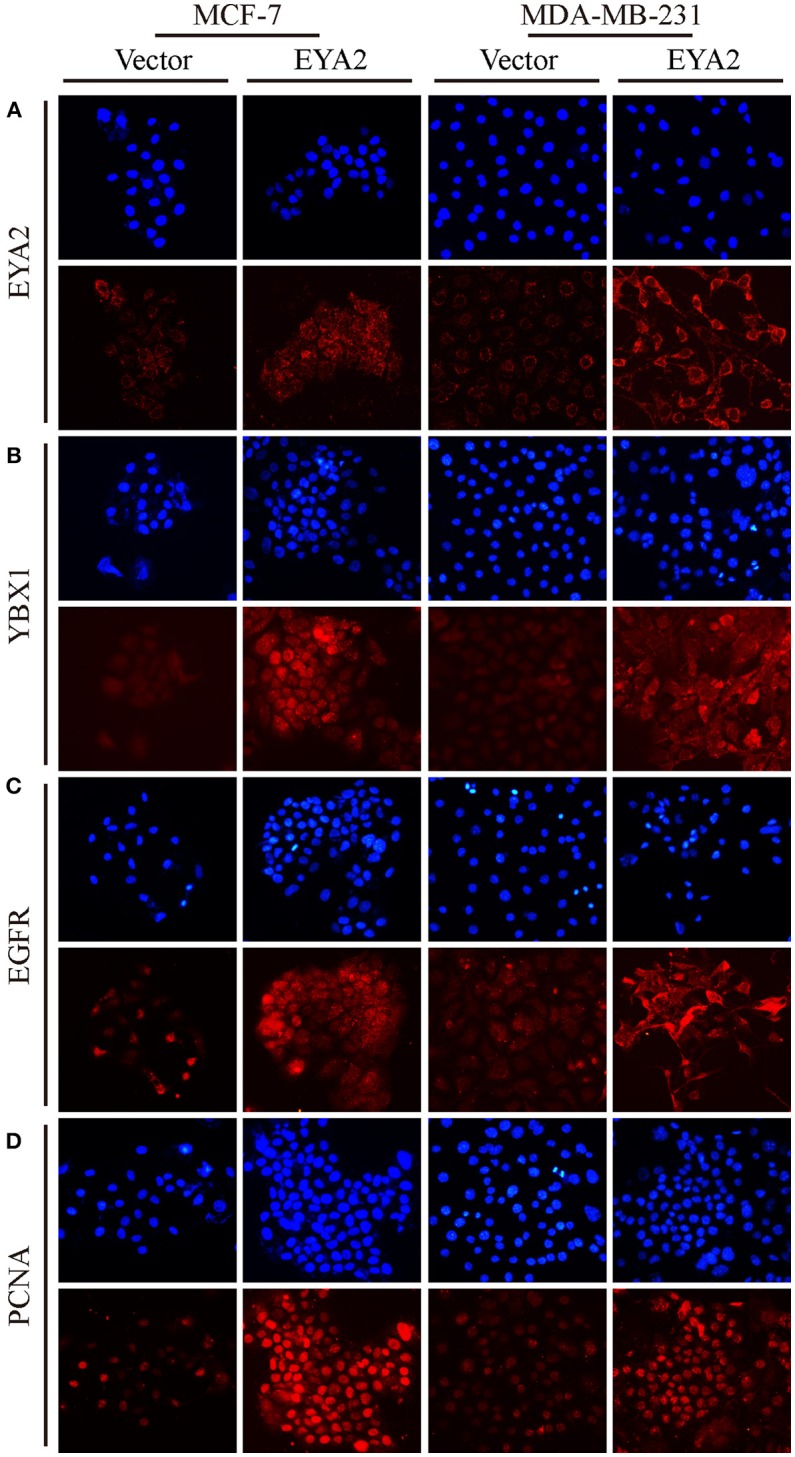
EYA2 over-expression up-regulates YBX1, EGFR, and PCNA. Cellular IF assay showed that EYA2 over-expression **(A)** enhanced the protein abundance of YBX1 **(B)**, EGFR **(C)** and PCNA **(D)** in both MCF-7 and MDA-MB-231 cells.

We also assessed the mRNA level of *EYA2* in distinct breast cancer cell types (42), showing that there was no remarkable difference of *EYA2* mRNA between luminal-type and basal-like cell lines (*p* = 0.666) (Supplementary Figure 1B). Therefore, two representative breast cancer cell lines (MCF-7 and MDA-MB-231) were selected to be transfected with *EYA2* or empty vectors. Colony-forming unit assays showed that both MCF-7 and MDA-MB-231 with EYA2 overexpression formed more clones with the same number of initiating cells in comparison with the controls (Figure 5A). EdU proliferation assay displayed that the ratio of proliferative cells was much higher among EYA2-overexpressing cancer cells than empty vector controls for both MCF-7 (*p* = 0.004) and MDA-MB-231 (*p* = 0.004) (Figure 5B). Western blotting showed that EYA2 overexpression induced the up-regulation of YBX1, EGFR, cyclin E, and PCNA in both these two breast cancer cell lines (Figure 5C). Cellular IF assay also showed that EYA2 overexpression (Figure 6A) enhanced the protein abundance of YBX1 (Figure 6B), EGFR (Figure 6C), and PCNA (Figure 6D) in both breast cancer cell lines.

### High EYA2 mRNA Predicted Poor Prognosis of Breast Cancer

**Figure 7:**
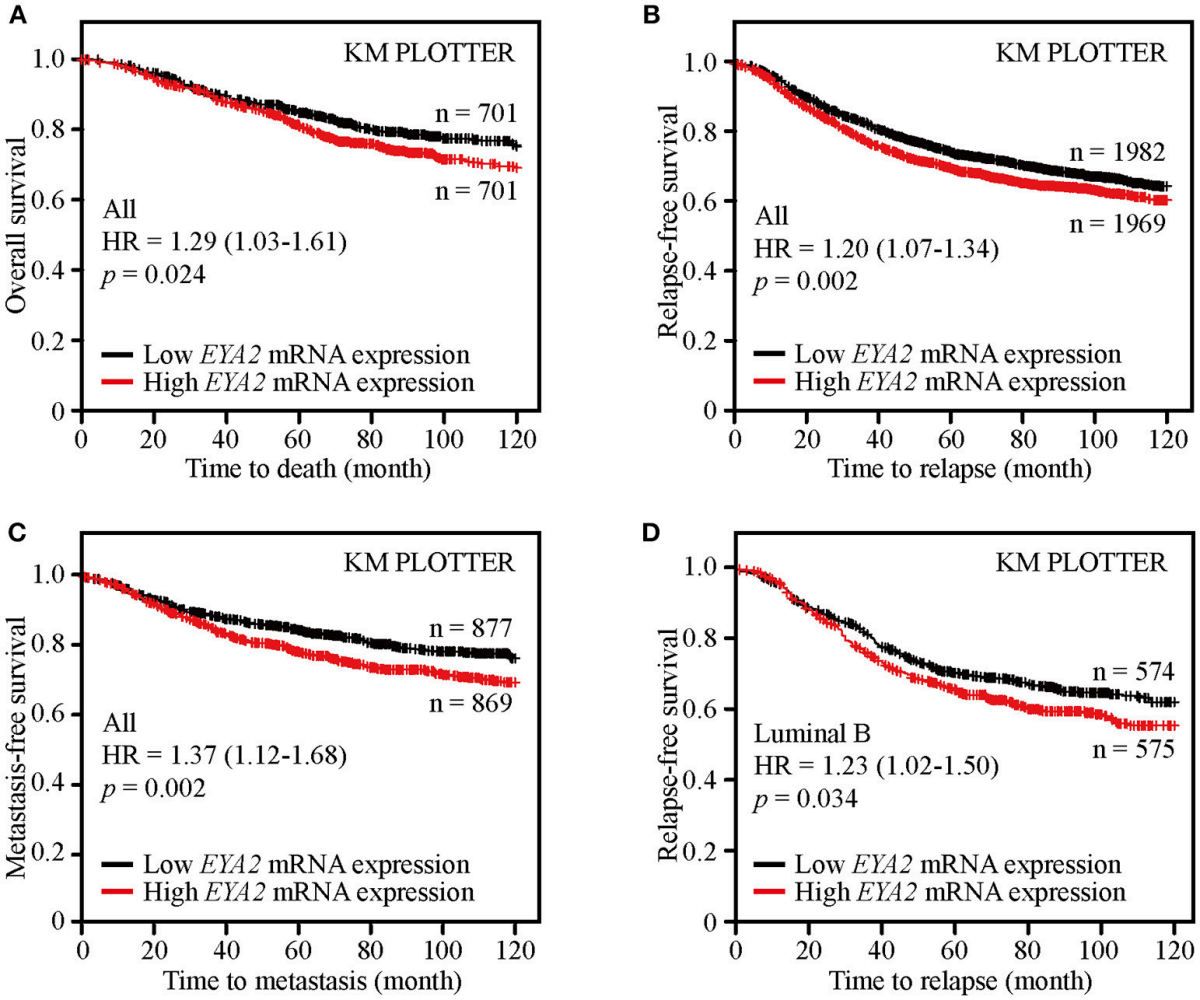
EYA2 was an unfavorable prognostic element for breast cancer patients. KM plotter analysis showed that higher *EYA2* mRNA expression was correlated with worse OS **(A)**, RFS **(B)** and MFS **(C)** at whole level, and patients with lower *EYA2* mRNA level enjoyed longer time free from tumor relapse among luminal B subgroup **(D)**.

Survival analysis of public available breast cancer datasets was conducted using the Kaplan-Meier Plotter platform to examine the clinical significance of *EYA2* in breast cancer. The results showed that patients with higher *EYA2* mRNA expression had worse OS [HR = 1.29 (1.03–1.61), *p* = 0.024; Figure 7A], RFS [HR = 1.20 (1.07–1.34), *p* = 0.002; Figure 7B] and MFS [HR = 1.37 (1.12–1.68), *p* = 0.002] (Figure 7C) among whole breast cancer population. Further subgroup analysis showed that higher *EYA2* mRNA level was correlated with worse RFS among luminal B subgroup [HR = 1.23 (1.02–1.50), *p* = 0.034; Figure 7D]. However, there was no statistically significant association between *EYA2* mRNA and clinical outcomes in basal-like breast cancer patients, including OS [HR = 1.49 (0.91–2.45), *p* = 0.11], RFS [HR = 1.04 (0.81–1.34), *p* = 0.75], and MFS [HR = 1.52 (0.91–2.55), *p* = 0.11].

## Discussion

EYA family was firstly identified as a key regulator for proper eye development in *Drosophila* (11). EYA2 has been implied in tumorigenesis and progression of some cancer types (14). Our results indicated that EYA2 was closely associated with tumor grade and molecular subtypes of breast cancer. Cellular experiments revealed that EYA2 promoted proliferation of breast cancer cell lines. Survival analysis based on the public database indicated that *EYA2* was an unfavorable prognostic element. Surprisingly, our analysis of GEO datasets showed that *EYA2* mRNA was dramatically lower in breast cancer tissues than normal breast. However, previous study indicated that EYA2 protein abundance increased in breast cancer tissues (43). This inverse tendency of EYA2 at mRNA and protein levels may arise from the complex regulatory processes from mRNA to protein.

Our results showed that *EYA2* mRNA was higher in grade 3 breast tumors than grade 1–2 cancer, indicating that EYA2 was correlated with poor-differentiation in breast carcinoma. FOXA1 is a marker for luminal epithelium and plays pivotal roles in mammary duct formation (44). Our correlation analysis displayed that *EYA2* was inversely associated with *FOXA1*, which further supported the correlation between high *EYA2* expression and poor tumor differentiation.ER, PR and HER2 are critical pathological markers in breast cancer. According to the status of ER, PR, and HER2, this highly heterogeneous disease can be roughly classified into three major molecular subtypes, including luminal-type, HER2-enriched, and TNBC. Among these three major subtypes, luminal type accounts for the most part of breast cancer population with relatively better prognosis, while TNBC group show more progressively malignant manifestation with worse clinical outcomes. Our results displayed that EYA2 expression was higher in hormone receptor (HR)-negativebreast cancer tissues in comparison with HR-positive cancerous tissues, while *EYA2* mRNA was positively associated with HER2 expression. In addition, EYA2 expression was remarkably enhanced in TNBC in comparison with luminal-type. In accordance, correlation analysis showed that *EYA2* mRNA was inversely correlated with the mRNA levels of luminal markers *ESR1, PGR* and *FOXA1*, and positively associated with TNBC markers *KRT5, KRT6B*, and *EGFR*. In consistence, it was reported that EGFR positively regulated EYA2 through the regulation of microRNA-338-3P to promote breast tumor growth and metastasis (18).

EMT is a pivotal process for tumor invasion and metastasis. Our results indicated that *EYA2* was positively correlated with the mesenchymal marker *SNAI2* at mRNA level. Liang et al.'s work demonstrated that EYA2 promoted EMT in lung cancer (18). In pancreatic adenocarcinoma, stable overexpression of EYA2 up-regulated transforming growth factor-β (TGF-β) signaling which is an important inducer of EMT (21). In agreement with our finding, knockdown of *EYA2* antagonized the *SIX1*-induced TGF-β signaling, and partially restored epithelial properties with a decrease of the mesenchymal marker fibronectin in breast cancer MCF-7 cells (45).

CSCs are inherently endowed with potent self-renewal capacity, and contribute to tumor initiation and progression. In our study, we showed that *EYA2* was associated with CSCs markers *YBX1, KLF5*, and *SOX10* in breast tumor tissues, and EYA2 overexpression up-regulated YBX1 in breast cancer cell lines. In pancreatic cancer, EYA2 overexpression enhanced the level of stem cell marker CD133 (21). Silencing of EYA2 led to a decrease of cells with CD44+ and CD24– among MCF-7 cells with exogenous overexpression of SIX1, indicating that EYA2 was required for SIX1 in the enhancement of CSCs features (45).

Cancer cells are characterized with unlimited proliferation. Results of EdU cell proliferation assay indicated that EYA2 promoted proliferation of tumor cells. *EYA2* mRNA was positively associated with the proliferative marker *CCNE1*, and EYA2 overexpression enhanced the expression of EGFR and PCNA. Previous studies elaborated that EYA2 drove the proliferation in multiple cancer types, including breast cancer (43), lung cancer (16, 20), and astrocytoma (17). In lung cancer, EYA2 promoted tumor cell proliferation through microRNA-93-mediated inhibition of phosphatase and tension homolog (16). As a downstream target of microRNA-30a, EYA2 could boost the proliferation of breast cancer cells through driving G1/S cell cycle progression with up-regulation of cell cycle-related proteins cyclin A, cyclin D1, and cyclin E (43).

DACH and EYA are the key members of RDGN. There are feedback regulations between DACH and EYA in both physiological and pathological situations (11). DACH1 was reported to be an anti-tumor protein in breast cancer (46), while EYA2 served as a tumor-driving molecule in breast carcinoma (18). Our results indicated that *EYA2* was inversely associated with *DACH1* in breast cancer. The imbalance of DACH1 and EYA2 may contribute to tumor initiation and development.

In some cancers, EYA2 acted as a prognostic predictor (15, 19, 21, 45). Although EYA2 cannot serve as an independent prognostic biomarker, high EYA2 expression was correlated with poor prognosis for pancreatic cancer patients (21). Among patients with advanced ovarian cancer, higher EYA2 level was correlated with worse OS (19). In agreement with our analysis, Farabaugh et al. found that higher expression of EYA2 was associated with worse RFS, MFS, and disease-specific survival (DSS) among 295 patients with invasive breast cancer (45), and high expression of both SIX1 and EYA2 represent the type with the worst RFS, MFS, and DSS in comparison with another three types including SIX1^low^/EYA2^low^ (with the best prognosis), SIX1^high^/EYA2^low^ and SIX1^low^/EYA2^high^ (45).

In conclusion, EYA2 was significantly correlated with clinico-pathological features of breast cancer, including tumor differentiation and the status of ER, PR, and HER2, and it was enriched in TNBC tumors. Furthermore, *EYA2* mRNA was positively associated with markers of TNBC, EMT, and CSCs. Besides, ectopic expression of EYA2 promoted proliferation of breast cancer cells accompanied with the up-regulation of EGFR, cyclin E, and PCNA. Importantly, Kaplan-Meier analysis of public datasets showed that higher *EYA2* mRNA level predicted worse prognosis among breast cancer population. Taken together, EYA2 promoted malignant behavior of breast cancer.

## Author Contributions

HX performed experiments and data analysis, drafted the manuscript, and prepared the figures. MY, YJ, and WZ carried out immunohistochemistry analysis. KW designed experiments and revised the manuscript. All authors read and approved the final manuscript.

### Conflict of Interest Statement

The authors declare that the research was conducted in the absence of any commercial or financial relationships that could be construed as a potential conflict of interest.

## Abbreviations

RDGN: retinal determination gene network
DACH: dachshund
SIX: sine oculis
EYA: eyes absent
EMT: epithelial-mesenchymal transition
EGFR: epidermal growth factor receptor
GEO: gene expression omnibus
ER: estrogen receptor
PR: progesterone receptor
HER2: human epidermal growth factor receptor 2
IHC: immunohistochemistry
TMA: tissue microarray
TNBC: triple-negative breast cancer
CSCs: cancer stem cells
IF: immunofluorescence
OR: odds ratio
95% CIs: 95% confidence intervals
OS: overall survival
RFS: relapse-free survival
MFS: metastasis-free survival
HR: hazard ratio
FOXA1: forkhead box A1
KRT5: keratin 5
YBX1: Y-box binding protein 1
KLF5: kruppel like factor 5
SOX10: sex determining region Y-box 10
PCNA: proliferating cell nuclear antigen
PBS: phosphate buffered saline
SPSS: Statistical Product and Service Solutions
TGF-β: transforming growth factor-β
DSS: disease-specific survival.
