# Supplementary material for: EYA2 Correlates With Clinico-Pathological Features of Breast Cancer, Promotes Tumor Proliferation, and Predicts Poor Survival
Source: Front Oncol. 2019 Jan 29;9:26. doi: 10.3389/fonc.2019.00026 (PMC6361843; doi:10.3389/fonc.2019.00026)
Supplement: Supplementary file 1 [file Data_Sheet_1.PDF]

Supplementary Figure 1

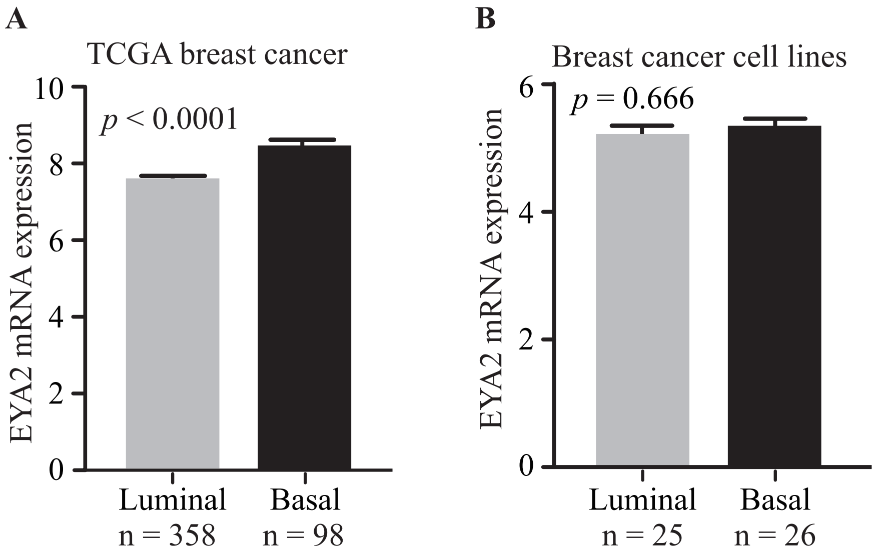

Supplementary Figure 1 The mRNA expression of *EYA2* in luminal-type and basal-like tumors. **(A)** Based on TCGA breast cancer data, *EYA2* mRNA level was significantly higher in basal-like breast cancer than luminal-type cancer tissues. **(B)** Based on breast cancer cell line data, there was no statistically significant difference of *EYA2* mRNA expression between luminal-type cancer cells and basal-like tumor cells.
